# Aging Attenuates Cardiac Contractility and Affects Therapeutic Consequences for Myocardial Infarction

**DOI:** 10.14336/AD.2019.0522

**Published:** 2020-03-09

**Authors:** Ming Dong, Ziyi Yang, Hongcheng Fang, Jiaqing Xiang, Cong Xu, Yanqing Zhou, Qianying Wu, Jie Liu

**Affiliations:** ^1^Department of Pathophysiology, Guangdong Key Laboratory of Genome Stability and Human Disease Prevention, Shenzhen University Health Science Center, Guangdong, China; ^2^Shenzhen Shajing Hospital, Affiliated of Guangzhou Medical University, Shenzhen, Guangdong, China

**Keywords:** aging, cardiac contractility, cardiac ischemia, ischemia and reperfusion injury, cardiac ischemia therapy

## Abstract

Cardiac function of the human heart changes with age. The age-related change of systolic function is subtle under normal conditions, but abrupt under stress or in a pathogenesis state. Aging decreases the cardiac tolerance to stress and increases susceptibility to ischemia, which caused by aging-induced Ca^2+^ transient impairment and metabolic dysfunction. The changes of contractility proteins and the relative molecules are in a non-linear fashion. Specifically, the expression and activation of cMLCK increase first then fall during ischemia and reperfusion (I/R). This change is responsible for the nonmonotonic contractility alteration in I/R which the underlying mechanism is still unclear. Contractility recovery in I/R is also attenuated by age. The age-related change in cardiac contractility influences the therapeutic effect and intervention timepoint. For most cardiac ischemia therapies, the therapeutic result in the elderly is not identical to the young. Anti-aging treatment has the potential to prevent the development of ischemic injury and improves cardiac function. In this review we discuss the mechanism underlying the contractility changes in the aged heart and age-induced ischemic injury. The potential mechanism underlying the increased susceptibility to ischemic injury in advanced age is highlighted. Furthermore, we discuss the effect of age and the administration time for intervention in cardiac ischemia therapies.

## 1. Introduction

Myocardial systolic and diastolic function diminish with age, contributing to high morbidity and mortality of cardiovascular diseases (CVDs) in the elderly [1]. Patients older than 65 years old usually have diastolic dysfunction who present a lower E/A and a longer left ventricular isovolumetric relaxation time (LVRT) than young adults [2]. In contrast, the systolic function change is not affirmative in the elderly because the ejection fraction (EF) and shortening fraction (FS) have yielded conflicting results [3]. More subtle changes in contractility, including decreased left ventricle end diastolic volume (LVEDV), peak systolic tissue velocity (S’), and left ventricular systolic pressure (LVSP), can be detected by more sensitive measurements in aging hearts. [4]. Aging elevates the susceptibility of the heart to ischemia and myocardial infarction (MI) [5]. According to a 2017 report, over 50% of ischemic heart diseases occur in people older than 70s. Cardiac contractility declines suddenly in post-ischemia infarction [6], which can induce heart failure and even cause death. The EF and FS drop significantly in response to cardiac stress in advanced age, which is implicated by the progressive degeneration and reduction in cardiac myocytes [7-9]. Beside changes in cellular level, a number of molecular alternation contribute to age related stress intolerance, including Ca^2+^ handling impairment, mitochondrial dysfunction, free radical accumulation, and alteration of myosin protein expression [1,10-12]. Recently, the crucial role of epigenetic alteration in the cardiac aging process has attracted much attention.

The therapeutic effect of ischemic cardiac dysfunction varies in old patients. Patients older than 80 have worse survival rate than 70-year-old patients after cardiac ischemic therapy, but the survival rate for patients who undergo coronary artery bypass grafting is not affected by age [13]. Early intervention for aging patients with ischemic heart disease will decrease the mortality rate [14]. The difference in therapeutic effects between the aged and young groups also existed following cell therapies. Aged mice with cardiac injuries that underwent cardiosphere-derived cell (CDCs) transplantation showed no improvement in cardiac function, while cardiac function was improved in the young [15]. Furthermore, the results of cardiac regeneration therapy for aging people is still in a matter of debate [16,17].

This review focuses on cardiac contractility changes and the underlying mechanism during aging. The potential mechanism of ischemia intolerance and the mechanism leading to a acute contractility dysfunction after ischemia in aging patients are also discussed. Finally, an overview of cardiac ischemic therapies and the effect of aging on those therapies are also provided in this review.

## 2. Cardiac contractility impaired in aging population.

Cardiac aging is accompanied by complex morphological and functional changes, involving compensatory left ventricular wall thickening to cardiomyocyte senescence and number loss [18]. The overall shape of the heart changes from elliptical to spheroid, which associate with cardiac wall stress and overall contractile efficiency [8]. Age-associated cardiac remodeling also implicates extracellular matrix (ECM) alterations. An aged cardiac ECM has 2% more of collagen content than a young cardiac ECM [19-21]. Excessive collagen and augmented collagen crosslinks lead to an age-related fibrosis and result in a myocardial contractility dysfunction including cardiac sarcopenia, and cardiac stiffness [19,22,23]

Previously, EF and FS are the main indices to evaluate cardiac contractility; however, EF and FS alterations in advanced age are disputable. Fiechter *et al.* [4] reported a positive relation between EF and age, measuredby magnetic resonance. Ruan *et al.* [3] and Ranson et al.[24] showed constant EF in elderly, but others demonstrated a decrease in EF with aging [25]. The gender ratio, race, and level of physical exercise were all different in the aforementioned studies, which may be a reason for the variable EF results. A preserved EF in early aging is hypothetically caused by enlargement of LVEDV or compensatory thickening of the left ventricular wall [26]. Therefore, EF alteration is unable to fully describe the contractility changes in the aging heart. More precise indicators are demanded to evaluate the subtle systolic functional changes. Global LV longitudinal strain (LS) and peak S’ decrease in hearts have been confirmed to be age-related [27-29]. A subdued LS primarily causes a declination of systolic blood pressure in the old [24]. A decrease in the LVSP and an elevation in left ventricle end diastolic pressure (LVDP) are obtained in old mice by hemodynamic measurements [30]. Precise measurement of cardiac contractility clarifies the aging-induced decline in contractility at a baseline physiological state.

Severe contractility dysfunction is easily identified under pathologic states with irregular cardiac contraction and decreased EF, FS, dp/dt, LVSP, and LVDP in the elderly [31-33]. Interestingly, there are some studies that have reported a non-linear decrease in cardiac contractility during I/R. The LVDP remains constant within 15 min of ischemia, whereas 50% decrease in mechanical function was noted when hearts are subjected to 20-25 min of ischemia. Moreover, 30 min of ischemia causes 100% inhibition of heart contractility without reperfusion [34]. Following reperfusion, systolic function recovers to normal within 5 min, but the LVDP continues to decrease and stabilizes at a level even lower than the ischemic state [34,35]. The speed and scope of recovery in the old heart are worse than in the young heart [36]. This phenomenon should cause a corresponding non-constant change on contractile myosin protein expression during IR, which is worthy of a detailed investigation.

## 3. Multiple mechanism regulate contractility of aged heart and increase susceptibility to ischemia.

### 3.1 Ca^2+^ transient

**Figure 1.**
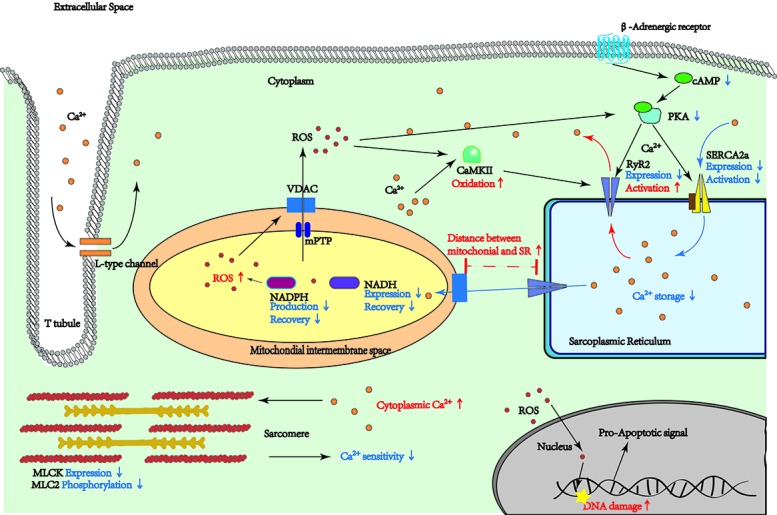
Regulatory pathways are affected by aging. This figure presents the pathways which are changed by aging in myocardium cells. The Arrows and lines in red are representing an increase by aging. The blue arrows are indicating a decrease in aging process.

Cardiac contraction is activated by a transient rise in intracellular free Ca^2+^. Ca^2+^ transient initiates L-type Ca^2+^ current influx and subsequently triggers Ca^2+^ release from the sarcoplasmic reticulum (SR) through the Ca^2+^ release channels and ryanodine receptors (RyRs) [37] (Fig. 1). The intracellular Ca^2+^ will activate the myofilament protein, then undergo reuptake back into the SR to achieve excitation-relaxation coupling [37]. Cardiomyocyte contraction, attenuated with age, relates to abnormal intracellular Ca^2+^ homeostasis, which is maintained by Ca^2+^ influx and SR Ca^2+^ storage [37,38]. One prominent change, involved the decay of Ca^2+^ transient, is significantly prolonged in aged cardiomyocytes [39]. Reduced expression of SR Ca^2+^ ATPase 2 (SERCA2a) and over-activation of RyRs are responsible for the prolonged SR Ca^2+^ transient in the aging heart. However, an opposite result of SERCA2a expression was recently reported on atrioventricular junction of 24-month-old Wistar rats [40]. This finding suggested to us that the Ca^2+^ transient might be different in each part of the heart during aging, which may involve aging contractility compensatory mechanisms. The overall increase in protein kinase A (PKA) and phospholamban (PLB) can also lead to SERCA2a dysfunction and slow Ca^2+^ re-uptake in 24-month old rats [37,41]. The delayed reuptake of Ca^2+^ diminishes SR Ca^2+^ storage during recovery and results in Ca^2+^ transient impairment; Schmidt *et al.* [42] and Del*et al.* [43] confirmed that +dp/dt improved in the senescence heart by overexpressing SERCA2a in rats; however, controversial results have been reported in clinical trials. [44-47] Although there are positive results in patients with end-stage HF by treating with SERCA2a carried by adeno-associated virus serotyp1 (AAV1) vectors (average age, 51 years) [44,45], no significant improvement in cardiac function by treating with the same method on patients who were on average 60 years old [46]. Hence, the therapeutic effects of SERCA2a potentially be age depended, but the limited efficiency of energy generation in aged hearts is one of the most possible reasons for this poor clinical outcome, since activity of SERCA2a largely relies on ATP generation and hydrolysis. Moreover, the effect of overexpression of SERCA2a might be different between natural aging and pathologic states. Expression of RyR, the major protein involved in SR Ca^2+^ release, is reported to have an age-associated reduction [27]; however the RyRs are over-activated by over-oxidized CaMKII and PKA in aged hearts, resulting in an enhanced diastolic Ca^2+^ leak and decreased SR Ca^2+^ storage [48]. Together with reduced expression of SERCA2, the aged heart has delayed and weaker peak contractions at higher stimulation frequencies [37] and finally causes contractile dysfunction [49,50]. Interestingly, the cAMP/PKA activation pathway is attenuated in aging heart which suppose to reduce the RyR activation. However excessive ROS in advanced age contribute most to oxidization of CaMKII and PKA. Kim *et al.*[51] rebuilt Ca^2+^ homeostasis and repaired RyR function by inhibiting ROS production in cardiomyocytes. Therefore, the attenuated recovery of Ca2+ homeostasis by ROS-induced over activated RyR is one of the potential reasons for AVV1/SERCA2a therapy inefficiency.

### 3.2 Mitochondrial dysfunction

Reduction of ATP production in the aging heart, which is caused by mitochondrial dysfunction, was observed in 2005 [52]. The cardiac contractility requires ATP turnover. The age-related change of metabolism attenuates cardiac contractile function [53]. Mitochondrial NADH level significantly declines in old myocytes after 10 min of continuous stimulation, while young myocytes remain constant [53]. Furthermore, although the contractile properties are comparable in young and old hearts at rest, aged hearts have a greater declination on peak shortening than young hearts with continuous stimulation [53]. This provide a explanation of excise intolerant for the old that NADH recovery efficiency might play a key role.

Excessive endogenous ROS caused by mitochondrial dysfunction is the main source of oxidative stress, which is related to multiple age-related cardiac morphologic changes, such as cardiac fibrosis, cell apoptosis, DNA damage, and cardiac remodeling [54]. Fernandez-Sanz *et al.* [55] demonstrated that weaken mitochondrial-SR interaction in aging cardiomyocytes is the cause of excessive ROS. Fernandez-Sanz *et al.* [55]observed an increased distance between SR and mitochondria during aging. The bridge between the mitochondrial outer membrane anion channel (VDAC) and RyR is disrupted in aging, leading to reduced Ca^2+^ uptake in mitochondria [55]. And this disruption will depress the production of NADPH and induce accumulation of ROS inside mitochondria. Then, mitochondrial permeability transition pore (mPTP) activity is enhanced by ROS-damaged VDAC, leading to a mitochondrial Ca^2+^ overload [56]. Over-activated mPTP allows ROS to cross through the mitochondrial membrane and release into the cytoplasm. Endogenous ROS modifies ion channels and Ca^2+^ coupling proteins, such as CaMKII and RyR2, resulting into abnormal excitation-contraction coupling. Treating the aged heart with MitoTEMPO, a ROS expression suppressor, increase contractile performance of senescence hearts [57]. Age-induced excessive ROS also promotes pro-inflammatory cytokine expression and stress kinases activation via the NF-κB, MAPK/p38, and related pathways to induce the low-grade chronic inflammation [58]. low-grade chronic inflammation environment in advanced age is related to increasing ischemia infarction size and contractile dysfunction [59]. The production of ROS is continuously after 3 h of I/R and can reduce Ca^2+^ sensitivity of myofilament proteins and then induce systolic dysfunction [60]. Excessive ROS not only elevates sensitivity to ischemia, but also leads to contractility dysfunction in normal senescence and pathologic states. Those studies strongly proved the theory that mitochondria dysfunction is the main cause of a series of functional changes during aging; however, evidence is lacking to prove that mitochondrial dysfunction initiates the aging process.

### 3.3 Myofilament protein disorder

The cMLCK/MLC-2 pathway directly regulates cardiac contractility in aging. Cardiac light chain kinase (cMLCK) is a Ca2+/calmodulin-activated protein kinase that phosphorylates the 20 kD regulatory light chain (MLC-2) of myosin [61,62]. The level of cMLCK protein has been shown to decrease in the aging heart, but the level of mRNA shows opposite tendency. Hence, post-transcriptional regulation may be the key regulatory mechanism for cMLCK. The level of MLC2 phosphorylation correspondingly drops in the aged heart [63]. Although diastolic dysfunction with the over-expression of MLC-2 has been shown to play a role in impairing relaxation with aging [64], no additional experiments have been conducted in extremely old animals, such as 30-month-old C57 mice. Some studies have chosen Fischer 344 X Brown Norway hybrid rats as aging model animals, but these rats are not under a normal state of senility. In cardiac ischemia injury, MLCK and phosphorylated MLC-2 decrease after cardiac ischemia [65]. However, Zhang et al observed an opposite results after I/R injury [66]. Although, the reason of those controversial results is still unclear, these findings potentially give a reason for the inconstant systolic change during ischemia. Moreover, Gao *et al.*[67] demonstrated that cMLCK over-expression has a protective effect in cell contraction and Ca^2+^ sensitivity during I/R. Treating cardiomyocytes with rhNRG-1, a growth factor inducing MLCK expression, can improve the EF% and FS% in murine hearts with cardiac I/R injury [68]. Old MLCK knockout mice show attenuated recovery of cardiac systolic function and severe cardiac remodeling after ischemia compared with null and young mice [69], so aging-induced loss of cMLCK expression and activation may contribute to the prolonged recovery in ischemia injury.

### 3.4 Noncoding RNA alteration

Accumulating evidence has shown that miRNA, small non-coding RNAs regulating mRNA translation, is an important regulator of the cardiac aging process and susceptibility to ischemia. Previously, miRNA-34a, miR-1, and miR-133a were shown to accelerate cardiac cell death and promote cardiac aging [70-72]. Recently, Lyu *et al.* [73] indicated that miR-29 accumulates in cardiac cells with increasing oxidative stress and decreased EF, followed by suppressed histone 4 lysine 20 trimethylation (H4K20me3) expression in mice [73]. miR-21 and miR-22 both increase in aging heart tissue to promote cardiac hypertrophy and fibrosis. Function of those altered miRNA in aging related myocardial infarction is poorly understood. Interestingly, the senescent cells are found to drive extracellular vesicles with 4-fold higher miRNAs per cell than normal cells [74]. The miRNA content is changed in extracellular vesicles with increasing age. The age-related miRNA changes in hypothalamic stem/progenitor cell-derived exosomes (htNSC-derived exosomes) have been shown to be the key to attenuating the speed of aging [74]. Although the detailed mechanism of exosomal miRNA is still elusive, those studies provide a new perspective to investigate the aging process.

Long non-coding RNAs (lncRNAs) also regulate mRNA expression in aging. LncRNA MALAT1 and MIAT regulate the aging process by controlling the cell cycle [75]. LncRNA H19 expression decreases with aging and promotes inflammatory cytokine production [76]. Notably, the lncRNA and miRNA interaction plays a crucial role in cardiac MI injury. LncRNA acts as a miRNA sponge to adhere miRNA and inhibit miRNA functions. LncRNA APF interacts with miRNA-188-3p which enhances cardiac autophagy and induces infarction size. Both miRNAs and lncRNAs are largely altered in advanced age hearts, but there are few reports about the influence of the lncRNA and miRNA interaction in the aging process. The interaction may be important to age-induced vulnerability to ischemia.

### 3.5 Histone modification alternation

Histone modification recently is found to play a crucial role in cardiac aging. Histone modification-related proteins, such as histone deacetylases (HDACs), are closely related to regulation of cardiac contractility. HDAC6 is associated with a-tubulin degradation [77] and pressure overload or chronic angiotensin II signaling [78], thus leading to systolic dysfunction. The mitral inflow velocity (E/A) and septal mitral annulus velocity (E’/A’) improve in aged mice with HDAC inhibitor administration [79]. An increase DNA methylation is observed in aging and regarded as a biological clock to assess the progression of senescense [80]. The epigenetic age acceleration elevates CVD mortality risk by 20% [81]. Recently, studies involving premature aging syndromes have uncovered another potential mechanism for aging-induced systolic impairment. A study focusing on Hutchinson-Gilford progeria syndrome (HGPS) revealed that a LMNA mutation is up-regulated in the aging process. Interestingly, the LMNA encoding protein, improper lamin A (progerin), is also found in normal aging [82,83]. Progerin can lead to DNA damage and nuclear defects [84]. Recently, normal aging related to up-regulation of progerin has been shown to evolve in dilated cardiomyopathy. High-level expression of progerin occurs in the dilated cardiomyopathy hearts of patients and is accompanied by declining EF, LVEDD, and LVESD. A linear negative correlation between EF and progerin mRNA level has been reported [85]. This finding shed light on lamin A function in cardiac remodeling during aging systolic dysfunction; however, the molecular mechanism underlying lamin A regulation of cardiac contraction is still undefined.

## 4. MI treatments targeting contractility related manner are affected by aging

### 4.1 Therapy targeting ROS and Ca^2+^ channels

ROS, as a therapeutic target, has been shown to suppress the cardiac aging process and to improve cardiac I/R injury. Roshni *et al.* [86] reported that aged cardiac stem/progenitor cells rejuvenate into a younger phenotype by decreasing ROS generation. Eliminating the excessive ROS in aged hearts and restoring mitochondrial function improve I/R injury. The over-expression of tissue inhibitor of metalloproteinases 3 (TIMP3) attenuates ROS production and ROS-induced MAPK activation, thereby decreases I/R-induced cardiac apoptosis and infarction size [87].

Meanwhile, ROS production and Ca^2+^ transient are closely interdependent. The ER Ca^2+^ leaking is mediated by the XO/ROS/mPTP pathway. Thus, inhibiting xanthine oxidase activity has been shown to reduce ROS production and restore cardiac ability in MI. SERCA2a function and Ca^2+^ transient are enhanced after administering a xanthine oxidase inhibitor [88]. Therefore, XO/ROS/mPTP pathway could potentially be a therapeutic target for aging induced AMI which may have a better therapeutic efficiency than the AVV1/SERCA2a therapy in the old patients. Low-level laser is also able to restore mitochondrial function and reduce oxidative stress. Systolic dysfunction was improved and infarct size was reduced by low-level laser in the early stage of MI. Interestingly, but no effect on ischemia-induced end-stage heart failure [89].

Compared to ROS therapy, regulation of Ca^2+^ transient is more effective in improving heart failure. Transient receptor potential vanilloid 4 (TRPV4), an ion channel involved in Ca^2+^ homeostasis, improves contractility by over-expression in both young and aged MI mice [90]. Both contractility function and cardiac remodeling in ischemia-induced heart failure are improved by over-expression of PSEN1, which induces the production of RyR2 in cardiomyocytes [91]. However, RyR2-related therapy may be similar to AVV1/SERCA2a therapy which is closely related with age, since the activation condition of these two channels are largely changed by aging. A combination therapy of RyR2/SERCA2a therapy and ROS elimination might improve therapeutic effects on the old. Therefore, further experiments for both Ca^2+^-channel-dependent therapy and ROS-related therapy on aged MI animal models are warranted.

### 4.2 Therapeutic effect of miRNA in exosomes are found in stem cell therapies

Stem cell therapies have been studied for a long time to mend MI. Cardiac regeneration could be initiated by transplantation or eliciting innate cardiomyocyte proliferation. Non-cardiomyocytes are used, such as mesenchymal stem cells (MSCs), bone marrow-derived mononuclear cells/mesenchymal stem cells (BM-MCs/MSCs), and pluripotent stem cells, as transplant material [92]. In a study of autologous MSC transplantation, results from aged recipients were not as encouraging as the results from the young, because aged donor cells exhibited a clearly lower survival rate and 2-fold less production response to ischemia [16]. Furthermore, the young recipients had grater regenerative responses compare to the old recipients [16]. Davis *et al.* [101] also demonstrated that the therapeutic potential of cardiac progenitor cells (CPCs) is age-dependent. This finding verified that age for recipient and donor cells could be an important contributor to cell-based therapy. However, in clinical trials the effect of aging in cell-based therapy is still elusive. In an MSC transplantation trial there was no significant impairment in old patients [17]. Not only the reduced absolute scar size from baseline to 1 year post-stem cell injection were not differ by age [17], but also both the > 60-year-old and < 60-year-old groups had no significant improve in EF.

Exosomes of cardiosphere-derived cells (CDCs) and MSCs, nano-extracellular particles carrying protein and RNAs from host cells, are both found to improve cardiac function after acute and chronic myocardial infarction [93,94]. Exosomes were applied to the infarction area by injection after 4 weeks of surgery in pigs. The myocardial infarction size shrank and the LVEF increased by treatment with intramyocardial exosomes [95]. Another study of CDC exosome was applied after I/R to 8-12-week-old Wistar rats. CDC exosomes still successfully preserved LVEF in the injured heart and reduced the infarction size [96]. Exosomal miRNAs have been shown to be the core agents in exosome therapy. The beneficial effect of miRNA-146a, which is down-expressed in cardiovascular aging, has been shown have protective effect on MI injury [70,97]. miR-181b in the CDC exosome is able to protect cardiac function and reduce infarction size by regulating macrophage polarization [98]. miR-181b has also been shown to be decreased in the aging aorta and induce cardiac stiffness. Therefore, studying the exosomal miRNAs of stem cells may lead to a deeper understanding on the function and mechanism of miRNAs in MI pathology.

### 4.3 Histone acetylate is a therapeutic target for both aging and aging-induced MI

HDAC inhibitors have recently been shown to effectively improve cardiac function and to reduce infarction size. Applying trichostatin A (TSA) and SAHA, FDA-proved HDAC pan-inhibitors, during reperfusion reduces infarction size and improves FS% in both mice and rabbits [99]. Interestingly, the TSA is not as effective as previously reported by Sverre et al when it is applied before surgery [100]. It can be concluded that the effect of HDAC cardiac inhibitors is affected by the drug application time point. Selective class I HDACs inhibitorwas injected 24 h and 1 h before ligation surgery. The cardiac dp/dt_max_ and developed pressure were well-preserved compared to the vehicle group, but still lower than control [100]. Furthermore, Hikmet Nural-Guvener^111^ reported that mocetinostat, a class I and IV HDAC inhibitor, is able to increase LVEF and +dp/dt in ischemia-induced heart failure and decrease infarction size. Since the crucial work of HDAC in the cardiac aging process was reported, HDAC inhibitors have recently been shown to have a promising effect on anti-aging [101]. Therefore, aging may minimally affect the drug effectivity of HDAC inhibitors in mending injured hearts. Histone acetyltransferases (HATs) are responsible for acetylate histonereversal by HDAC. Inhibition of HATs is effective in cardiac MI therapy by inhibiting activity of p300, a potential therapy of heart failure from years ago. Curcumin is a commonly used p300 inhibitor in myocardial infarction treatment. There are several studies that have reported the beneficial effect of reducing cardiac infarction by pre-treatment with curcumin, a p300 inhibitor. Liu *et al.* [102] applied curcumin in different doses (10, 20, or 30?mg/kg/d) to SD rats 20 days before anterior descending coronary artery ligation surgery. The infarction size of the treated group was decreased approximately 2.5-fold compared to the I/R group. Furthermore, STAT3 phosphorylation was enhanced by curcumin administration which indicted that JAK2/STAT3 signal pathway might be a crucial downstream target for p300/HAT regulation. Xiao *et al.* [103] reported that curcumin (100 mg/kg/day orally) pre-treatment reduced cardiac fibrosis by down-regulating SIRT1. Recently, the curcumin analogs (demethoxycurcumin [DMC] and bisdemethoxycurcumin [BDMC]) were both shown to be as effective as curcumin in preventing cardiac hypertrophy [103]. Moreover, combination therapy with curcumin and enalapril has been shown to be more effective than curcumin alone in improving LVFS in myocardial infarction [104].

## 5. Anti-aging therapy could potentially improve the ischemic injury

Healthy aging will cause approximately the loss of one-third of cardiomyocytes in the human heart. Senescence of long-lived differentiated myocardial cells may induce apoptosis to consequent on severe impairment following myocardial infarction [105]. Therefore, anti-senescence could potentially alleviate ischemic damage in the aging heart by eliminating the myocyte apoptosis. The first anti-senescence drugs (senolytics) were inverted in 2015. Dasatinib plus quercetin, the first anti-senescence drugs combination, killed senescent cells by targeting the senescence signals in cells, for instance ephrins (EFNB1 or 3), PI3Kδ, p21, BCL-xL, and plasminogen-activated inhibitor-2, to prolong the lifespan of mice [106]. This combination also enhances physical functioning in old mice. Irradiation-treated adipose cells were transplanted into 8-month-old mice, which led to global senescence in multiple organs, including the heart. After treatment of old mice with senolytics, the walking speed, food intake, and daily activities were improved [107]. But the cardiac function was not assessed in this research.

Rapamycin, another found senolytic, is able to rejuvenate aging stem cells by targeting the mTOR/PI3K pathway. Resveratrol is also a well-known drug that benefits cell function by targeting mitochondrial biogenesis. The combination of these two drugs has been shown to prevent cell senescence and improve cardiac output following myocardial infarction [108]. These studies have shown the therapeutic potential of anti-senescence and cardiac rejuvenation to cure myocardial infarction or prevent aging-induced ischemia susceptibility; however, the underlying mechanism and side effects are still unknown.

## 6. Conclusion

Contractility dysfunction is not as apparent as diastolic dysfunction in heathy aging. But, systolic dysfunction is stimulated by cardiac stress or a pathogenic state in advanced age [39]. Complicated interaction in oxidative stress, Ca^2+^ transient, myofilament reaction and epigenetic regulation are responsible for the poor recovery and adjustment on contractility after ischemia in advanced age. However, the aging process is not regulated by an isolated molecular pathway. Interestingly, the cell organelle interaction, specifically the SR-mitochondrial interaction, are involved into the myocardial function impairment in advanced age. This study reminded us the significant engagement of inter-organelle communication in cardiac aging process which indeed is worthy for more attention.

Epigenetic regulation has also presented a crucial role in both cardiac aging and pathology of MI. Independent study of miRNAs and lncRNAs in aging process has reveal their regulatory mechanisms, but the role of interaction between those non-coding RNAs in aging and aging-induced MI are still poorly investigated. Further more, Ounzain *et al.* [109] has found the close correlation between cardiac specified lncRNA, H3K4me1 and H3K27Ac in MI. However, due to limited study, we are not able to further discuss about the significance of this correlation in aging induced MI. Further investigation in histone modification and RNA interactions will provide a good point of penetration to understand pathogenic mechanisms for the cardiovascular diseases and new therapeutic target for clinical application in future.

The effectiveness of stem cell therapies is always influenced by aging-related environmental changes. For example, ageing-induced low grade systematic inflammation cause poor survival rate of injected stem cells [110,111]. Similarly, stem cell-derived exosome therapies are also largely limited by recipient cell senescence which causes a deteriorated cell proliferation ability and a weaken cardiac myocyte performance [112]. Therefore, effect of those therapies could be enhanced by anti-aging pretreatment.

Surprisingly, administration time point during I/R is also crucial to the therapeutic effect of a therapy. TSA has clearly shown a difference between pre-treatment and post-reperfusion administration. To optimize the administration timepoint of each therapy, molecular and cellular alteration during I/R need to be further investigated. Previous study has reveal a non-linear change of proteome in duration of I/R [34]. Therefore, the corresponding alteration should also appear at the transcriptional and translational level. To further investigate those alterations could potentially lead us to more specified therapeutic targets for each stage of I/R.

## 7. Summary

Aging researches on MI are largely limited by the difficulties of animal model establish, because the high mortality of old animals after surgery. Therefore, the different pathological differences between young and old are not explicit. The mitochondrial disfunction, Ca^2+^ transient impairment and myofilament alternations has been closely studied in aging researches, but epigenetic regulation, especially histone modification, is still full of unknowns. A research gap on the function and effect of ageing-induced histone modification in aging-induced ischemic injury is worthy to pay more attention. Further study on the aging induced molecular and cellular alterations will provide scientific basis for precision medicine for the elderly and novel biomarkers for cardiac aging and aging related cardiac dysfunction in the future. The effect of aging on clinical myocardial infarction treatments are also in an urgent need. Those studies could help to improve the therapeutically effects and provide appropriate therapeutic regimens for the elderly.
